# A Progressive Ratio Task with Costly Resets Reveals Adaptive Effort-Delay Trade-Offs

**DOI:** 10.1523/ENEURO.0258-25.2025

**Published:** 2025-11-12

**Authors:** Zeena M. G. Rivera, Kimberly Guerrero Leon, Megan Cervera, Berlin Aguayo, Alicia Izquierdo, Andrew M. Wikenheiser

**Affiliations:** ^1^Department of Psychology, UCLA, Los Angeles, California 90095; ^2^The Brain Research Institute, UCLA, Los Angeles, California 90095; ^3^Integrative Center for Learning and Memory, UCLA, Los Angeles, California 90095; ^4^Integrative Center for Addictions, UCLA, Los Angeles, California 90095

**Keywords:** decision-making, foraging, motivation, progressive ratio

## Abstract

The progressive ratio (PR) schedule is a popular test of motivation. Despite its popularity, the PR task hinges on a low-dimensional behavioral readout—breakpoint or the maximum work requirement subjects are willing to complete before abandoning the task. Here, we show that with a simple modification, the PR task can be transformed into an optimization problem reminiscent of the patch-leaving foraging scenario, which has been analyzed extensively by behavioral ecologists, psychologists, and neuroscientists. In the PR with reset (PRR) task, male and female rats performed the PR task on one lever but could press a second lever to reset the current ratio requirement back to its lowest value at the cost of enduring a reset delay, during which both levers were retracted. Rats used the reset lever adaptively on the PRR task, and their ratio reset decisions were sensitive to the cost of the reset delay. We derived an approach for computing the optimal bout length—the number of rewards to earn before pressing the reset lever that produces the greatest long-term rate of reward—and found that rats flexibly changed their behavior to approximate the optimal strategy. However, rats showed a systematic bias for bout lengths that exceeded the optimal length, an effect reminiscent of “overharvesting” in patch-leaving tasks. The PRR task thus represents a novel means of testing how rats adapt to the cost–benefit structure of the environment in a way that connects deeply to the broader literature on associative learning and optimal foraging theory.

## Significance Statement

The progressive ratio with resets (PRR) task offers a novel approach to studying how animals weigh time and effort when making decisions. Unlike traditional progressive ratio tasks, PRR allows rats to reset the escalating ratio requirement at the cost of a delay. Rats used the reset option adaptively, working longer before resetting when the reset delay was long. We developed a normative model of PRR task performance and found that rats’ behavior was correlated with model-derived predictions but that rats consistently overworked, completing more ratios per bout of work than the optimal strategy. The PRR task offers a more ecologically valid alternative to breakpoint-based measures of motivation, with potential applications in modeling impulsivity and prefrontal function in health and disease.

## Introduction

Understanding how animals weigh the costs and benefits of their actions is a central goal in decision neuroscience. The progressive ratio (PR) schedule has long been a key tool for approaching this question (Hodos, 1961). In PR sessions, subjects perform an instrumental response to earn reinforcers, and the ratio requirement increases with each reinforcer earned. Eventually, the ratio requirement becomes so costly that animals cease working. Breakpoint ratio—the highest successfully completed ratio—reflects the maximum effort cost subjects are willing to pay to obtain the reinforcer. The PR schedule has been used across species to investigate motivation for a range of reinforcers, including drugs of abuse, social interactions, and access to preferred activities (Stafford et al., 1998; Jerome and Sturmey, 2008). PR methods have also been successfully adapted to incorporate a choice between high- and low-value outcomes (i.e., highly palatable food vs standard chow) with different effort requirements (Salamone et al., 1991; Hart et al., 2017, 2018, 2020; Hart and Izquierdo, 2019).

Despite its widespread use, the PR approach has several notable limitations. Foremost, the influence of effort and delay on decision-making are confounded, as higher ratio requirements take longer to complete. This makes it difficult to tell whether sensitivity to effort or delay ultimately leads subjects to abandon the task. The limited response options available in PR also make it difficult for animals to explore different behavioral strategies—to lever press or not to lever press is the question posed on PR, which leaves little room for investigating the dynamics of behavior. Finally, as a behavioral readout, breakpoint is low-dimensional and lacks sensitivity—it both fails to capture known determinants of behavior and is also influenced by a number of nonmotivational factors (Aberman et al., 1998; Stafford et al., 1998; Cagniard et al., 2006; Yohn et al., 2015; Chen et al., 2022). These issues are less problematic when PR is used simply as a test of how motivated subjects are to earn a reinforcer, but they severely limit the utility of PR as a test of decision-making.

Here, we introduce a new variation on the classic PR task by incorporating a strategically meaningful opportunity for subjects to control the effort requirement. In the PR with reset (PRR) task, rats can choose to press a second lever at any time to reset the ratio requirement back to one, at the cost of enduring a reset delay during which no reinforcers can be earned. The PRR task mimics key features of patch-leaving foraging problems, in which animals harvest diminishing resources in their current patch but may relocate—at a cost—to start anew elsewhere (MacArthur and Pianka, 1966; Charnov, 1976; Stephens and Krebs, 1986; Stephens, 2008). Similarly, ratio resets in the PRR task segment behavior into discrete bouts of work, each beginning with a low-effort, high-reward phase and ending when the rat chooses to incur the reset delay.

While a previous study investigated the impact of a cost-free reset option on PR performance (Hurwitz and Harzem, 1968), the present PRR method yokes the ratio reset to a costly delay, which substantially alters the economic structure of the task. Resets allow subjects to limit their effort expenditure, but resetting too frequently imposes a substantial opportunity cost, as a large fraction of each session is consumed by the delay. The PRR task thereby recasts PR as an optimization problem, in which judicious use of the reset option affords subjects the opportunity to earn a much higher rate of reward compared with standard PR. The PRR task also creates a richer decision space, allowing subjects to express a wider range of behavioral strategies for cost–benefit decision-making.

Here, we characterized rats’ behavioral performance on the standard PR and PRR tasks. We found that rats used the reset option in an adaptive, cost-sensitive manner, and reset decisions were largely similar between male and female rats. We describe an approach for determining the optimal behavioral strategy on the PRR task and show that rats’ performance correlated with the optimal strategy. However, rats also showed a systematic bias to work for too long before resetting the ratio.

## Materials and Methods

### Subjects

Adult Long–Evans rats (*n* = 24, 12 females) were sourced from Inotiv. The first cohort was aged postnatal day (PND) 96 and the second cohort was aged PND 260 (*n* = 6, three females) at the start of the experiment. Males weighed an average of 340 g, and females weighed 210 g. Rats were initially pair-housed and then individually housed prior to food restriction and behavioral testing. Rats were housed in a vivarium with a reverse light cycle (12 h dark/light cycle, lights on at 6 P.M.) and controlled temperature and humidity conditions (room temperature 22–24°C).

On arrival to the vivarium, rats were allowed to acclimate for at least 3 d in their home cage with no experimenter interference. Rats were then handled for 10 min daily for 5 d prior to behavioral testing. Rats were food restricted to no less than 85% of their free-feeding weight prior to behavioral testing and were maintained at a consistent weight throughout testing. All procedures were conducted in accordance with the recommendations in the Guide for the Care and Use of Laboratory Animals of the National Institutes of Health and with the approval of the University of California, Los Angeles Chancellor's Animal Research Committee.

### Pretraining

Behavioral sessions were conducted in operant boxes (Med Associates, model MED-008-D1) outfitted with two retractable levers, two cue lights (one above each lever), and a food magazine. Rats first completed magazine training where they were given 15 min to consume five sucrose pellets (45 mg, rodent purified diet, F0021-J, Bioserv) manually placed in the magazine. Next, rats moved on to lever-press training; in each lever-press training session, responses on one lever were reinforced on an FR1 schedule. When rats earned at least 30 pellets within a 30 min session, they would then undergo lever-press training sessions (also on an FR1 schedule) of the opposite lever until reaching the same criterion. Rats rarely took >2 sessions to reach criterion on each lever.

### Behavioral testing

Following pretraining, rats were tested in daily 45 min sessions on the PR or PRR behavioral tasks. Two versions of the PRR task were tested, one with a 10 s reset delay (PRR-10) and one with a 60 s reset delay (PRR-60).

In PR sessions, rats earned food by pressing the active lever, which began with a ratio requirement of one and incremented by one with each reinforcer earned. Whenever the rat reached the required number of lever presses for the current ratio, the light above the PR lever illuminated, and one food pellet would be dispensed into the magazine. The PR lever was inactive until the light turned off, which was prompted by the rat entering the magazine port to collect the pellet. Lever presses on either the inactive lever or on the active lever when the light above was illuminated were logged but did not count toward the ratio requirement.

In PRR task sessions, rats earned food by pressing the active lever, which was reinforced following the same reward schedule as in PR sessions. However, rats could press the second lever (the reset lever) at any time to initiate a ratio reset. Pressing the reset lever caused both levers to retract from the box until the reset delay passed, after which both levers reinserted into the box and the ratio requirement on the active lever was reset to one. The reset delay was 10 s in PRR-10 sessions and 60 s in PRR-60 sessions.

Rats were tested on a random sequence of PR, PRR-10, and PRR-60 sessions. Rats were given at least two practice sessions on each task before data collection began. For each rat, the identity of the active (left or right) was consistent across all tasks and testing sessions. Assignment of the active lever was counterbalanced across subjects. After every test session, the operant boxes were cleaned using Vimoba 128 and rats were fed supplemental standard rodent chow, with the amount titrated based on the amount of food they earned performing the task.

One female rat successfully completed the pretraining sequence but only pressed the reset lever four times total during all PR and PRR sessions; because this rat pressed the reset lever multiple orders of magnitude less frequently than all other rats, her data were excluded from subsequent analysis.

### Data analysis

All analyses were conducted using MATLAB (MathWorks). We used linear mixed-effect models (MATLAB function, *fitlme*) to assess the impact of the task type and other predictors on behavioral outcomes. All mixed-effect models included subject-specific intercepts to account for repeated measures (Yu et al., 2022). For analyses involving the task type (PR, PRR-10, PRR-60), the task was treated as a categorical fixed effect with PR assigned to be the reference level, such that model coefficients for PRR-10 and PRR-60 reflect differences relative to the standard PR task. Where appropriate, additional fixed effects (i.e., reset delay, sex, active lever-press rate) were included in models. The table accompanying each model reports the full model specification, coefficient estimates, and statistics.

To test for pairwise differences between conditions, we computed estimated marginal means from the fitted models by holding predictors at their average values and estimating means for each task condition. Post hoc corrections for multiple comparisons were performed using the Bonferroni–Holm correction.

### Modeling

To evaluate rats’ choices on the PRR task, we applied a normative modeling framework grounded in foraging theory (Stephens and Krebs, 1986; Lendrem, 2012). Bouts of lever pressing were treated analogously to patch residence times, while the ratio resets imposed a fixed “travel” cost. The goal of the model was to identify the bout length (i.e., the number of rewards to earn before resetting the ratio) that maximized the net reward rate, defined as the number of reinforcers earned divided by the time taken to earn them, including both work time and reset delays.

Because we could not directly measure the caloric expenditure associated with lever pressing or otherwise account for effort costs in physical units, we used each rat's session-specific active-lever–press rate as a proxy for energetic investment. This allowed us to estimate how long a rat would take to complete a bout of work of any length. A step-by-step derivation of the relevant equations—including those for computing bout duration and bout-level reward rate (*R*_bout_)—is provided in the main text. We used numerical simulations to determine the bout length that maximized *R*_bout_ for each session, given the rat's observed press rate and the session's reset-delay duration. These model-derived optimal bout lengths served as session-specific benchmarks for comparing with observed behavior. This normative modeling approach does not presume any particular psychological or neural mechanism for generating behavior. Rather, it establishes a rational performance baseline, allowing us to assess whether rats behaved in a manner that was reward-maximizing given their own press rates and the structure of the task.

When computing *R*_bout_, we used a handling time (the amount of time it takes rats to collect and consume a reinforcer after completing the ratio requirement) value of 2 s. While we could have measured handling time experimentally, simulations revealed that handling time influenced the numerical value of *R*_bout_ but did not affect the optimal bout length under a wide range of plausible handling-time values. This is because handling time adds a fixed delay for each reward, and the total handling-time penalty for each bout therefore scales linearly with the number of rewards earned. In contrast, the reset delay is a lump-sum cost paid once per bout, no matter how many rewards are earned. As a result, optimizing bout length involves balancing a single, bout-level penalty (the reset delay) against the marginal benefits of staying longer. Since handling time affects all bouts proportionally to their length, it shifts the entire reward rate curve downward without changing the point at which the marginal benefit of continuing to work falls below the cost of resetting.

### Code accessibility

All data and code are freely accessible at https://zenodo.org/records/15577773.

## Results

**Figure 1.**
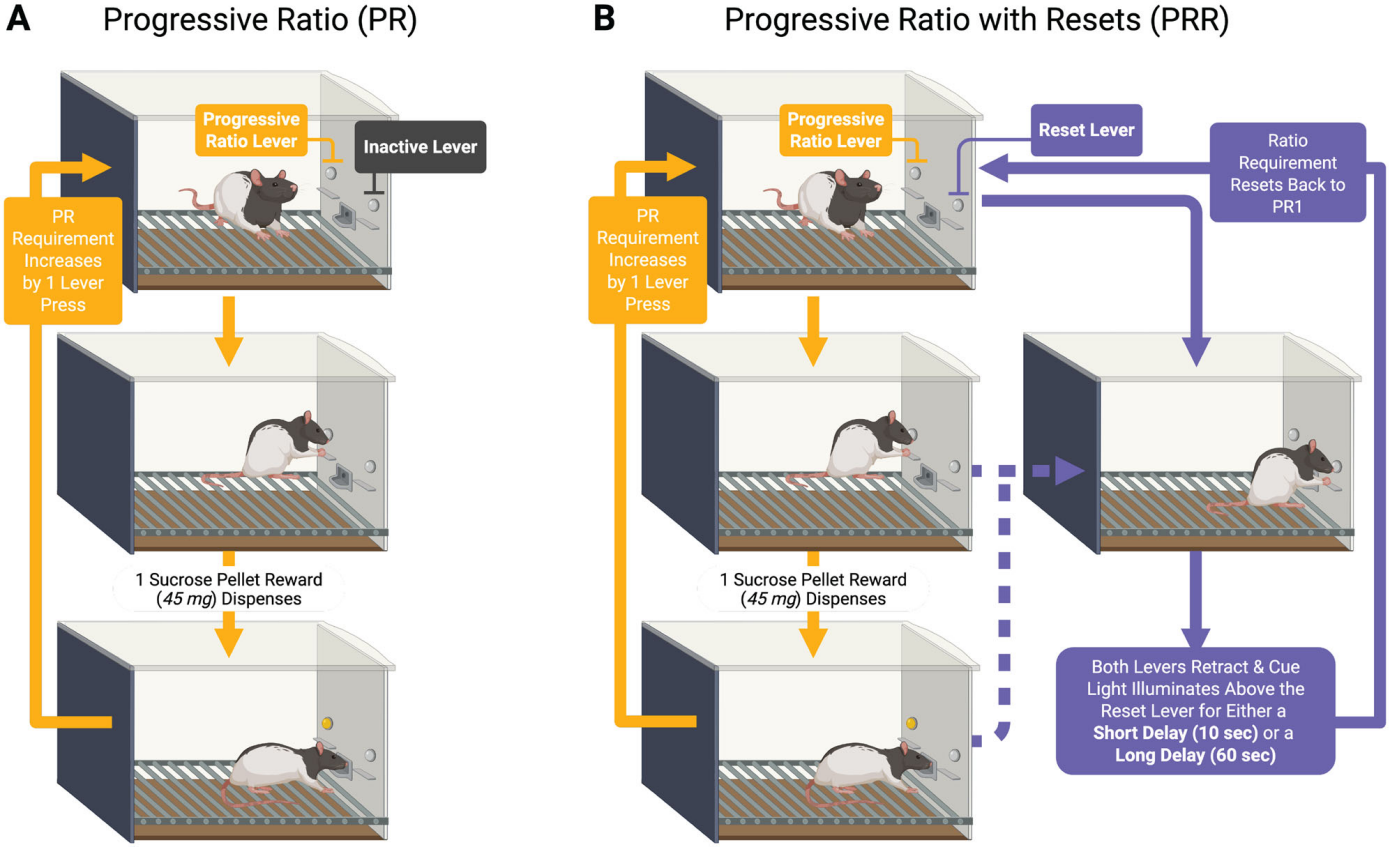
PR and PRR task structure. Rats were tested on standard PR sessions (***A***) and two versions of a PR task in which a second lever reset the ratio requirement after a fixed reset delay (***B***). In PRR-10 sessions, the reset delay was 10 s, while in PRR-60 sessions, the reset delay was 60 s.

We tested rats (*n* = 24, 12 females) on standard PR (Fig. 1*A*) and two PRR task versions (Fig. 1*B*). In the PR task, rats encountered two levers in a standard operant chamber. The first press on the active lever resulted in delivery of one sucrose pellet. Each subsequent pellet required one additional press of the active lever. Presses on the second lever had no programmed consequences during PR sessions. In PRR task sessions, rats again encountered two levers. Pressing the active lever earned reward under the same escalating ratio schedule used in the PR task. However, in PRR sessions, rats could press a second lever which caused both levers to retract for a fixed delay, after which they re-extended and the response requirement on the active lever was reset to one. The reset-delay length was fixed within PRR sessions. We tested rats on short reset delays of 10 s (PRR-10) and long reset delays of 60 s (PRR-60). Rats performed one of the three tasks daily in a random order. Task was not cued in any way, requiring rats to sample the reset lever to determine whether they were performing a PR, PRR-10, or PRR-60 session.

### Ratio resets segmented PRR task performance into discrete bouts of work

**Figure 2.**
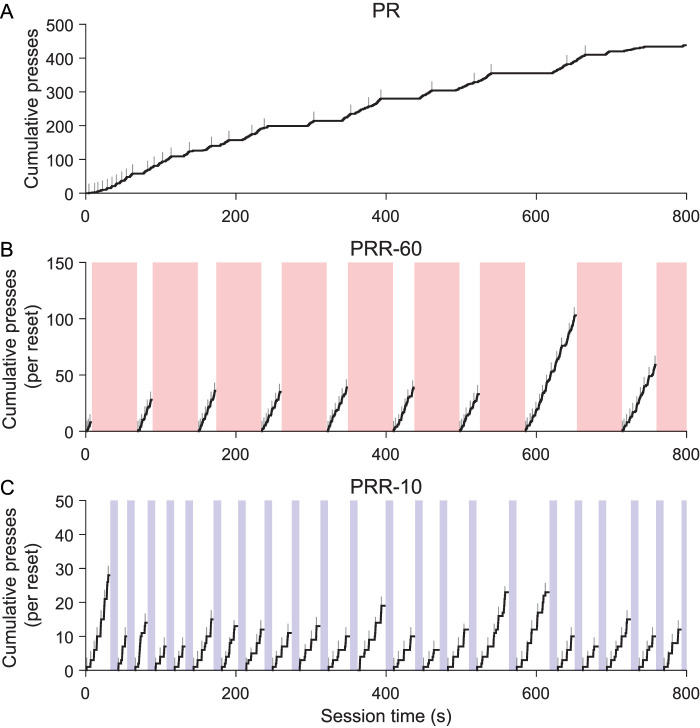
Ratio resets segmented PRR task performance into bouts of work. Cumulative records of active lever presses are plotted for the first 800 s of three example sessions. Gray tick marks indicate reward delivery times. ***A***, In PR task sessions, rats earned reinforcers quickly at the beginning of sessions, but the reward rate decreased as the ratio requirement progressed. Example record from Session 2 of PR. In PRR sessions (***B***, ***C***), lever pressing was segmented into bouts by ratio resets, which resulted in the reset delay (shaded regions). Example records of Session 2 PRR-60 (***B***) and Session 2 PRR-10 (***C***). Because reset presses returned the ratio requirement to one, cumulative records for PRR-10 and PRR-60 sessions were computed separately for each bout of work between successive ratio resets. Note the very different *y*-axes between the three example sessions.

Example cumulative records (Fig. 2) show the general structure of behavior on the three tasks. In these plots, active lever presses increment the cumulative record, and gray tick marks indicate when the current ratio requirement was complete and reward was available in the magazine. Performance on an example PR session (Fig. 2*A*) was consistent with previous reports: rats worked steadily early in the session but tended to slow responding as the ratio requirement increased. On both PRR tasks, behavior was structured into bouts of active lever pressing punctuated by reset presses. Because the ratio requirement reset after each delay, we computed cumulative active presses separately for each bout of lever pressing to visualize how many rewards were earned between resets. The example sessions show that rats worked to higher ratios and earned more reward with each bout of work in PRR-60 sessions (Fig. 2*B*) compared with PRR-10 sessions (Fig. 2*C*).

**Figure 3.**
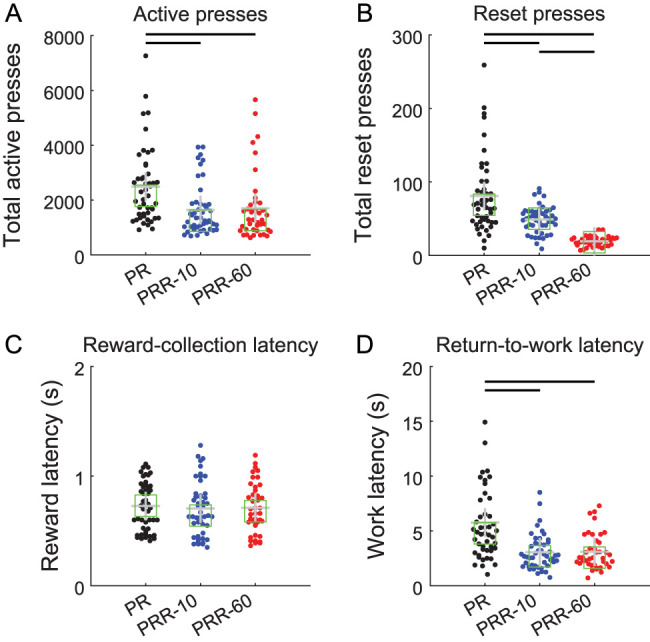
Rats used the reset lever in a cost-sensitive manner. ***A***, Rats made significantly more lever presses in PR sessions compared with PRR-10 and PRR-60. Each point represents total active presses from one testing session. Crosses mark the mean and squares mark the median of each group, while bars between groups denote significant pairwise differences as tested by post hoc contrasts (same conventions apply for panels ***B–D***). ***B***, Rats used the reset lever in a cost-sensitive way, pressing most frequently when it had no programmed benefit or cost in PR sessions, and pressing it least frequently in PRR-60 sessions when the reset delay was maximal. ***C***, Median reward–collection latency did not differ across the three tasks. ***D***, After earning a reinforcer, rats’ median latency to resume active lever pressing was longest for PR sessions and did not differ between PRR-10 and PRR-60 sessions.

Consistent with the example cumulative records, the number of active presses per session was significantly higher for PR compared with PRR-10 (*β*_PRR-10_ = −848; *p* = 7.39 × 10^−13^) and PRR-60 (*β*_PRR-60_ = −827; *p* = 1.40 × 10^−11^; mixed-effect model; Table 1) sessions. Pairwise post hoc comparisons found significantly more active presses in PR sessions compared with PRR-10 (*p* = 2.22 × 10^−12^) and PRR-60 (*p* = 2.81 × 10^−11^) sessions. Active lever presses did not differ between PRR-10 and PRR-60 sessions (*p* = 0.85; linear contrasts from mixed-effect model with Bonferroni–Holm correction for multiple comparisons; Fig. 3*A*).

**Table 1.** A mixed-effect model testing the influence of task on total active lever presses per session

Model structure: active lever presses ∼ task + (1 | Rat ID)					
Effect	Estimate	SE	95% CI		*p*
			LL	UL	
Intercept	2,466.39	226.68	2,017.96	2,914.82	4.87 × 10^−20^
PRR-10	−848.48	106.66	−1,059.48	−637.47	**7.39 × 10^−13^**
PRR-60	−827.37	111.69	−1,048.32	−606.42	**1.40 × 10^−11^**

LL, lower limit of confidence interval; UL, upper limit of confidence interval; SE, standard error. The task (PR, PRR-10, PRR-60) was coded as a categorical variable, and PR served as the reference level for comparison. A unique intercept was fit for each subject to account for the repeated-measure structure of the data. Bold *p*-values denote *p* < 0.05.

Rats used the reset lever sparingly in all three tasks, pressing it approximately an order of magnitude less frequently than the active lever (Fig. 3*B*). A mixed-effect model (Table 2) found significantly fewer reset presses in PRR-10 (*β*_PRR-10_ = −32; *p* = 9.84 × 10^−7^) and PRR-60 (*β*_PRR-60_ = −63; *p* = 5.71 × 10^−17^) sessions compared with PR sessions. Post hoc contrasts of estimated marginal means confirmed significant differences in the number of reset presses between all pairs of task types: PR versus PRR-10 (*p* = 1.97 × 10^−6^), PR versus PRR-60 (*p* = 1.71 × 10^−16^), and PRR-10 versus PRR-60 (*p* = 5.89 × 10^−6^). This pattern is consistent with delay-cost-sensitive use of the reset lever: reset presses were most frequent in PR (where the reset lever had no programmed consequences) and decreased in frequency as the cost of resetting the ratio increased in PRR-10 and PRR-60.

**Table 2.** A mixed-effect model testing the influence of task on total reset lever presses per sessions

Model structure: reset lever presses ∼ task + (1 | Rat ID)					
Effect	Estimate	SE	95% CI		*p*
			LL	UL	
Intercept	80.62	5.04	70.64	90.60	1.44 × 10^−32^
PRR-10	−31.85	6.20	−44.12	−19.59	**9.84 × 10^−7^**
PRR−60	−62.51	6.48	−75.32	−49.69	**5.71 × 10^−17^**

LL, lower limit of confidence interval; UL, upper limit of confidence interval; SE, standard error. The task (PR, PRR-10, PRR-60) was coded as a categorical variable, and PR served as the reference level for comparison. A unique intercept was fit for each subject to account for the repeated-measure structure of the data. Bold *p*-values denote *p* < 0.05.

Reward collection latency (the duration between a reward becoming available and the rat retrieving it; Fig. 3*C*) did not differ between PR and PRR-10 (*β*_PRR-10_ = −0.02; *p* = 0.11; mixed-effect model; Table 3). The model detected a small difference in reward latency between PR and PRR-60 (*β*_PRR-60_ = −0.03; *p* = 0.04), which did not survive post hoc comparison of marginal means (*p* = 0.56; linear contrast from mixed-effect model). Return-to-work latency, the duration between reward collection, and the resumption of active lever pressing (Fig. 3*D*) were significantly greater on PR compared with PRR-10 (*β*_PRR-10_ = −2.66; *p* = 1.69 × 10^−9^) and PRR-60 (*β*_PRR-60_ = −2.67; *p* = 6.84 × 10^−9^; Table 4), and these differences were confirmed by post hoc contrasts (PR vs PRR-10, *p* = 5.07 × 10^−9^; PR vs PRR-60, *p* = 1.37 × 10^−8^). This likely arises because rats worked to much higher ratios on PR and postreinforcer pauses are known to scale with the upcoming ratio requirement (Griffiths and Thompson, 1973; Killeen et al., 2009; Skinner, 2019). Post hoc testing indicated no difference in work latency between PRR-10 and PRR-60 (*p* = 0.99).

**Table 3.** A mixed-effect model testing the influence of task on the median reward latency of each sessions

Model structure: reward latency ∼ task + (1 | Rat ID)					
Effect	Estimate	SE	95% CI		*p*
			LL	UL	
Intercept	0.74	0.05	0.64	0.83	5.58 × 10^−32^
PRR-10	−0.02	0.01	−0.05	0.01	0.11
PRR-60	−0.03	0.02	−0.06	0.00	**3.71 × 10^−2^**

LL, lower limit of confidence interval; UL, upper limit of confidence interval; SE, standard error. We computed the latency between ratio completion and reward collection for every reward and calculated the median latency for each session. The task (PR, PRR-10, PRR-60) was coded as a categorical variable, and PR served as the reference level for comparison. A unique intercept was fit for each subject to account for the repeated-measure structure of the data. Bold *p*-values denote *p* < 0.05.

**Table 4.** A mixed-effect model testing the influence of task on return-to-work latency

Model structure: return-to-work latency ∼ task + (1| Rat ID)					
Effect	Estimate	SE	95% CI		*p*
			LL	UL	
Intercept	5.82	0.44	4.94	6.69	7.69 × 10^−26^
PRR-10	−2.67	0.41	−3.48	−1.85	**1.69 × 10^−9^**
PRR-60	−2.67	0.43	−3.52	−1.82	**6.84 × 10^−9^**

LL, lower limit of confidence interval; UL, upper limit of confidence interval; SE, standard error. For each reward rats earned, we measured the duration between reward collection and the next active-lever press and calculated the median latency for each session. The task (PR, PRR-10, PRR-60) was coded as a categorical variable, and PR served as the reference level for comparison. A unique intercept was fit for each subject to account for the repeated-measure structure of the data. Bold *p*-values denote *p* < 0.05.

### Rats worked longer as the reset delay increased

**Figure 4.**
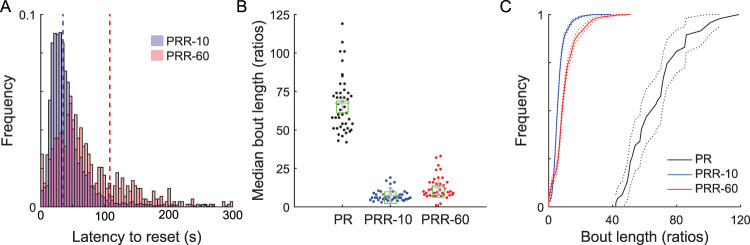
Rats work longer between resets when resets are more costly. ***A***, The average latency to reset the ratio was shorter in PRR-10 than PRR-60 sessions. Distributions depict all reset latencies from PRR-10 and PRR-60 sessions, and dashed vertical lines mark the mean of each distribution. ***B***, The median number of ratios rats completed during bouts of work in each session was greater in PR than PRR-10 and PRR-60. Each point shows median bout length from one session; crosses indicate the mean and boxes indicate the median of each distribution. See Extended Data Figure 4-1 for breakdown of these data by sex. ***C***, Cumulative distributions of all bout lengths from all sessions show that rats produced the longest bouts of work for PRR sessions, intermediate bout lengths for PRR-60 sessions, and the shortest bouts of work for PRR-10 sessions. Dotted lines depict the 95% confidence interval around each cumulative distribution function.

We next more carefully examined how rats used the reset lever on the PRR tasks. We first computed reset latency, the time between the insertion of levers (either at the beginning of the session or the end of a reset delay) and the next reset press. This measure captures how long rats tended to work on the active lever before resetting the ratio (Fig. 4*A*). In PRR-10, reset latency peaked at ∼25 s, while in PRR-60, reset latency peaked later, at ∼40 s, demonstrating that rats worked longer between reset presses when resets were costly. Notably, very short reset latencies (i.e., <10 s) were infrequent in both PRR tasks, suggesting that rats were sensitive to the consequences of pressing the reset lever and rarely reset the ratio successively without devoting some time to earning rewards.

10.1523/ENEURO.0258-25.2025.f4-1 Figure 4-1 **Reset delay affected bout length differently in male and female rats.** Median bout length for PRR sessions is plotted separately for male and female rats. Each point indicates the median bout length in one session, and error bars indicated the mean ± SEM. General patterns are similar across male and female rats with longer reset delays eliciting longer bouts of work. However, this effect was strong in male rats, leading to a significant interaction between sex and reset delay (Table 8). Download Figure 4-1, TIF file.

We next computed bout length, the median number of ratios rats completed during bouts of work in each session (Fig. 4*B*). Because the lack of a reset option rendered PR one long bout of work, we compared PRR bout length with PR breakpoint in a mixed-effect model (Table 5). Task significantly modulated bout length (*β*_PRR-10_ = −60; *p* = 7.34 × 10^−70^; *β*_PRR-60_ = −55; *p* = 2.25 × 10^−63^), and post hoc contrasts revealed significant pairwise differences between all tasks: PR versus PRR-10 (*p* = 2.20 × 10^−69^), PR versus PRR-60 (*p* = 4.50 × 10^−63^), and PRR-10 versus PRR-60 (*p* = 8.03 × 10^−3^). Cumulative distributions of all bout lengths from all sessions (Fig. 4*C*) showed the same pattern, with PRR-10 bouts being the shortest, PR bouts being the longest, and PRR-60 bouts intermediate to the other tasks.

**Table 5.** A mixed-effect model testing the influence of task on bout length

Model structure: bout length ∼ task + (1 | Rat ID)					
Effect	Estimate	SE	95% CI		*p*
			LL	UL	
Intercept	67.30	2.02	63.30	71.29	7.72 × 10^−66^
PRR-10	−59.83	1.66	−63.12	−56.55	**7.35 × 10^−70^**
PRR-60	−55.16	1.74	−58.59	−51.72	**2.25 × 10^−63^**

LL, lower limit of confidence interval; UL, upper limit of confidence interval; SE, standard error. Bout length was computed as the median number of rewards earned between reset presses for PRR-10 and PRR-60 sessions. For PR sessions, bout length was taken as the breakpoint ratio. The task (PR, PRR-10, PRR-60) was coded as a categorical variable, and PR served as the reference level for comparison. A unique intercept was fit for each subject to account for the repeated-measure structure of the data. Bold *p*-values denote *p* < 0.05.

### Reinforcer earnings varied with the cost structure of the tasks

The previous analyses have shown that PR and PRR tasks elicited different behavioral strategies, but do not address whether those strategies were effective. The number of reinforcers earned provides a summary measure of how effectively rats adapted to each task. Average earnings were greatest for PRR-10 (mean pellets/session = 328.4), intermediate for PRR-60 (mean pellets/session = 209.9), and lowest for PR (mean pellets/session = 67.4). A mixed-effect model (Table 6) found that total pellets was strongly affected by task, with rats earning significantly more pellets in PRR-10 (*β*_PRR-10_ = 261; *p* = 4.92 × 10^−8^) and PRR-60 (*β*_PRR-60_ = 143; *p* = 1.02 × 10^−56^) compared with standard PR. Post hoc contrasts confirmed that earnings differed significantly between all pairs of tasks: PR versus PRR-10 (*p* = 3.06 × 10^−56^), PR versus PRR-60 (*p* = 1.56 × 10^−28^), and PRR-10 versus PRR-60 (*p* = 7.55 × 10^−23^).

**Table 6.** A mixed-effect model testing the influence of task on total pellet earnings in each session

Model structure: total pellets ∼ task + (1 | Rat ID)					
Effect	Estimate	SE	95% CI		*p*
			LL	UL	
Intercept	65.53	11.32	43.14	87.91	4.92 × 10^−8^
PRR-10	261.45	9.42	242.81	280.08	**1.02 × 10^−56^**
PRR-60	142.74	9.86	123.23	162.24	**5.78 × 10^−29^**

LL, lower limit of confidence interval; UL, upper limit of confidence interval; SE, standard error. The task (PR, PRR-10, PRR-60) was coded as a categorical variable, and PR served as the reference level for comparison. A unique intercept was fit for each subject to account for the repeated-measure structure of the data. Bold *p*-values denote *p* < 0.05.

### Performance was correlated across PR and PRR tasks

We tested whether there was any consistency in how individual rats behaved across the three tasks. Average bout length was significantly correlated between all pairs of tasks (PRR-10 vs PRR-60, *r*_(24)_ = 0.92; *p* = 1.10 × 10^−10^; PRR-10 vs PR, *r*_(24)_ = 0.74; *p* = 3.30 × 10^−5^; PRR-60 vs PR; *r*_(24)_ = 0.77; *p* = 1.10 × 10^−5^; Pearson's correlation). That the performance of individual rats was correlated in this way suggests that all three tasks measured a similar form of cost-sensitivity and that inter-rat differences in this construct were stable across tasks.

### Limited evidence for sex differences in basic performance metrics

We next tested whether male and female rats performed any of the tasks differently from one another. On PRR-10 and PRR-60, the number of bouts of work that subjects completed in each session was affected by reset-delay length (*β* = −29.9; *p* = 2.52 × 10^−17^), but not by sex (*β* = 1.28; *p* = 0.80) or its interaction with reset delay (*β* = 1.26; *p* = 0.75; Table 7). Similarly, while bout length was affected by reset delay (*β* = 6.55; *p* = 1.83 × 10^−11^), there was no main effect of sex (*β* = −3.19; *p* = 0.10). Bout length, however, was significantly modulated by the interaction of sex and reset delay (*β* = −2.98; *p* = 0.01; Table 8). Inspection of these data (Fig. 4-1) revealed qualitatively similar patterns across male and female rats, with the significant interaction reflecting that the reset delay increased bout length in male rats more strongly than it did in female rats. In the PR task, breakpoint was not different between male and female rats (Table 9). There was no influence of reset-delay length, sex, or their interaction on reward latency in the PRR tasks (Table 10), nor was there an effect of sex on reward latency on PR (Table 11). Female rats showed greater return-to-work latencies on the PRR tasks (*β* = 1.51; *p* = 0.01), taking ∼2 s longer on average to resume working for the next ratio than male rats; neither reset delay or the interaction of reset delay and sex significantly affected work latency (Table 12). Similarly, female rats were slower by ∼3 s to resume work on PR (*β* = 4.42; *p* = 8.21 × 10^−6^; Table 13).

**Table 7.** A mixed-effect model testing the influence of sex on total work bouts in PRR sessions

Model structure: total work bouts ∼ sex + reset delay + sex × reset delay + (1 | Rat ID)					
Effect	Estimate	SE	95% CI		*p*
			LL	UL	
Intercept	48.09	3.66	40.80	55.38	6.05 × 10^−22^
Sex (“female” = 1)	1.28	5.17	−9.00	11.56	0.81
Reset delay (“60 s” = 1)	−29.99	2.80	−35.55	−24.42	**2.52 × 10^−17^**
Sex × reset delay	1.26	3.96	−6.61	9.14	0.75

LL, lower limit of confidence interval; UL, upper limit of confidence interval; SE, standard error. The number of work bouts was computed for each PRR session. PR sessions were not included in this analysis, as the lack of a ratio reset option meant each PR sessions consisted of a single bout of work. Sex was coded as a categorical variable (female = 1). Reset delay was coded as a categorical variable (60 s = 1). The interaction between sex and reset delay was included in the model to assess whether male and female rats were differentially affected by reset delay. A unique intercept was fit for each subject to account for the repeated-measure structure of the data. Bold *p*-values denote *p* < 0.05.

**Table 8.** A mixed-effect model testing the influence of sex on bout length in PRR sessions

Model structure: bout length ∼ sex + reset delay + sex × reset delay + (1 | Rat ID)					
Effect	Estimate	SE	95% CI		*p*
			LL	UL	
Intercept	9.11	1.30	6.53	11.70	5.76 × 10^−10^
Sex (“female” = 1)	−3.19	1.83	−6.84	0.46	**8.61 × 10^−2^**
Reset delay (“60 s” = 1)	6.55	0.84	4.88	8.23	**1.83 × 10^−11^**
Sex × reset delay	−2.98	1.19	−5.35	−0.61	**1.45 × 10^−2^**

LL, lower limit of confidence interval; UL, upper limit of confidence interval; SE, standard error. Sex was coded as a categorical variable (female = 1). Reset delay was coded as a categorical variable (60 s = 1). The interaction between sex and reset delay was included in the model to assess whether bout length was affected by reset delay differently in male and female rats. A unique intercept was fit for each subject to account for the repeated-measure structure of the data. Bold *p*-values denote *p* < 0.05.

**Table 9.** A mixed-effect model testing the influence of sex on PR breakpoint

Model structure: breakpoint ∼ sex + (1 | Rat ID)					
Effect	Estimate	SE	95% CI		*p*
			LL	UL	
Intercept	72.38	4.72	62.87	81.88	1.70 × 10^−19^
Sex (“female” = 1)	−10.79	6.66	−24.20	2.62	0.11

LL, lower limit of confidence interval; UL, upper limit of confidence interval; SE, standard error. Sex was coded as a categorical variable (female = 1). A unique intercept was fit for each subject to account for the repeated-measure structure of the data.

**Table 10.** A mixed-effect model testing the influence of sex on reward latency in PRR-10 and PRR-60 tasks

Model structure: PRR reward latency ∼ sex + reset delay + sex × reset delay + (1| Rat ID)					
Effect	Estimate	SE	95% CI		*p*
			LL	UL	
Intercept	0.67	0.07	0.53	0.81	2.39 × 10^−15^
Sex (“female” = 1)	0.09	0.10	−0.11	0.28	0.38
Reset delay (“60 s” = 1)	−0.01	0.02	−0.04	0.03	0.69
Sex × reset delay	−0.01	0.02	−0.06	0.04	0.81

LL, lower limit of confidence interval; UL, upper limit of confidence interval; SE, standard error. Sex was coded as a categorical variable (female = 1). Reset delay was coded as a categorical variable (60 s = 1). The interaction between sex and reset delay was included in the model to assess whether reward latency was affected by reset delay differently in male and female rats. A unique intercept was fit for each subject to account for the repeated-measure structure of the data.

**Table 11.** A mixed-effect model testing the influence of sex on PR reward latency

Model structure: PR reward latency ∼ sex + (1 | Rat ID)					
Effect	Estimate	SE	95% CI		*p*
			LL	UL	
Intercept	0.67	0.06	0.55	0.79	6.67 × 10^−15^
Sex (“female” = 1)	0.12	0.08	−0.05	0.29	0.16

LL, lower limit of confidence interval; UL, upper limit of confidence interval; SE, standard error. Sex was coded as a categorical variable (female = 1). A unique intercept was fit for each subject to account for the repeated-measure structure of the data.

**Table 12.** A mixed-effect model testing the influence of sex on PRR return-to-work latency

Model structure: PRR return-to-work latency ∼ sex + reset delay + sex × reset delay + (1| Rat ID)					
Effect	Estimate	SE	95% CI		*p*
			LL	UL	
Intercept	2.44	0.42	1.61	3.28	1.04 × 10^−7^
Sex (“female” = 1)	1.51	0.59	0.33	2.69	**1.25 × 10^−2^**
Reset delay (“60 s” = 1)	−0.14	0.17	−0.48	0.20	0.42
Sex × reset delay	0.13	0.24	−0.35	0.62	0.59

LL, lower limit of confidence interval; UL, upper limit of confidence interval; SE, standard error. Sex was coded as a categorical variable (female = 1). Reset delay was coded as a categorical variable (60 s = 1). The interaction between sex and reset delay was included in the model to assess whether return-to-work latency was affected by reset delay differently in male and female rats. A unique intercept was fit for each subject to account for the repeated-measure structure of the data. Bold *p*-values denote *p* < 0.05.

**Table 13.** A mixed-effect model testing the influence of sex on PR return-to-work latency

Model structure: PR return-to-work latency ∼ sex + (1 | Rat ID)					
Effect	Estimate	SE	95% CI		*p*
			LL	UL	
Intercept	3.51	0.63	2.25	4.77	1.22 × 10^−6^
Sex (“female” = 1)	4.42	0.88	2.65	6.18	**8.21 × 10^−6^**

LL, lower limit of confidence interval; UL, upper limit of confidence interval; SE, standard error. Sex was coded as a categorical variable (female = 1). A unique intercept was fit for each subject to account for the repeated-measure structure of the data. Bold *p*-values denote *p* < 0.05.

Collectively, these results suggest that male and female rats made decisions about how long to work and when to reset the ratio in a largely similar way. The observed sex difference in return-to-work latency might suggest small but measurable motivational differences between male and female rats, possibly due to female rats approaching satiety more quickly due to their smaller body size and therefore feeling less urgency to resume work quickly. On the other hand, the lack of sex difference in reward latencies suggests that male and female rats were similarly eager to collect and consume the pellets they earned.

### Modeling optimal PRR behavior

As noted in the introduction, the structure of the PRR tasks closely resembles the patch-leaving foraging problem. The PR schedule can be thought of as a depleting food resource, and resetting the ratio is analogous to traveling to a new, nondepleted patch. Optimality modeling has frequently been used to determine the optimal behavioral strategy in patch-foraging scenarios (MacArthur and Pianka, 1966; Emlen, 1968; Stephens and Krebs, 1986; Schoener, 1987), so we sought to apply this approach to the PRR tasks.

**Figure 5.**
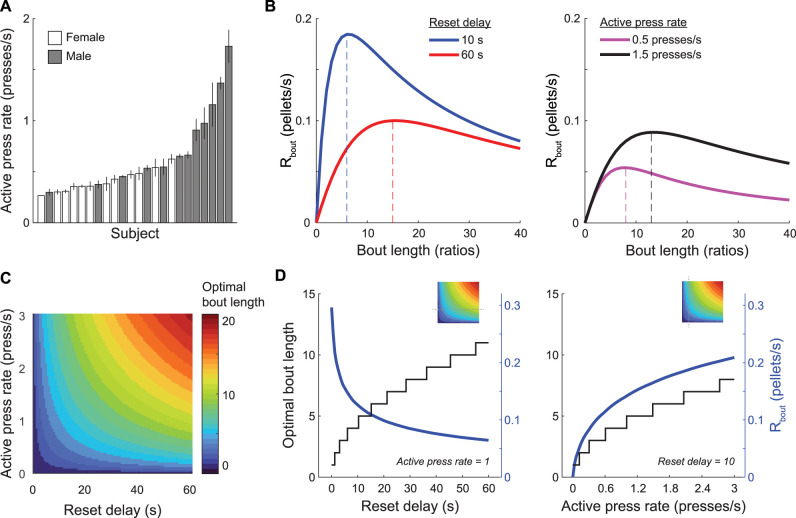
An optimality model for determining optimal PRR bout length. ***A***, Mean active-lever–press rate (±SEM) during PRR sessions is plotted separately for each rat in ascending order. There were consistent individual differences in active press rate across subjects. ***B***, *R*_bout_ (the rate of reward associated with working a particular bout length before resetting the ratio on the PRR task) is plotted for bout lengths varying from 0 to 40 rewards, for reset delays of 10 or 60 s (left) and for fast or slow active press rates (right). *R*_bout_ was maximal at longer bout lengths under the 60 s reset-delay condition. Faster lever-press rates shifted the optimal bout length to larger values. ***C***, The heat map depicts optimal bout length over a range of reset-delay lengths and active press rates. ***D***, Two slices through the heat map are plotted to more clearly show the effect of reset delay (left) and active press rate (right) on optimal bout length and *R*_bout_. Insets show the location of the line plots in ***D*** relative to the heat map in ***C***.

Optimal behavior on the PRR tasks should maximize net energy intake—that is, calories earned less the energetic costs of earning them. Finding the optimal strategy in the space of actual energy gains is not feasible for the PRR tasks because the number of calories burned per lever press likely varies in complex ways with the basal metabolic rates of individual rats and the speed or timing of lever presses. While we cannot easily determine how costly each lever press was, we can use the active press rate in each session as a proxy for how much energy rats chose to expend on the task. The average active press rate varied across sessions, and there were individual differences across subjects (Fig. 5*A*). Armed with an average active-lever–press rate (*p*_active_) for each session, the time to complete any ratio requirement (*n*) can be estimated as follows:

$$t_{n}=\frac{n}{\;p_{\mathrm{active}}}.$$

Similarly, the time to complete a bout of work of length *n* ratios can be computed as follows:

$$t_{\mathrm{bout}}=t_{\mathrm{reset}}+n\times t_{\mathrm{handle}}+t_{1}+\;t_{2}+t_{3}+\ldots t_{n},$$

where *t*_handle_ is the amount of time it takes rats to collect and consume each pellet after earning it (handling time, following the conventions of foraging theory) and *t*_reset_ is the reset delay. Including *t*_reset_ in the calculation ensures that the time penalty for resetting the ratio is accounted for in each bout, as bouts end with a reset press by definition. The number of reinforcers earned per bout of work is simply *n*, so the rate of reward associated with a bout of work length *n* is computed as follows:

$$R_{\mathrm{bout}}=\frac{n}{t_{\mathrm{bout}}}.$$

Figure 5*B* shows how *R*_bout_ changed as bout length and active press rate were varied. *R*_bout_ was computed separately for the 10 and 60 s reset-delay conditions, the active lever-press rates was fixed at one press per second, and the handling time was fixed at 2 s (Fig. 5*B*, left). *R*_bout_ peaked at different bout lengths for the two reset delays, with reward rate maximal at a bout length of 6 ratios when the reset delay was 10 s and a bout length of 15 ratios when the reset delay was 60 s. When reset delay was fixed at 60 s and active-lever–press rate was varied (Fig. 5*B*, right), the bout length that maximized the reward rate increased with the active-lever–press rate.

For a given rate of active lever pressing and a known reset delay, the optimal bout length is that which maximizes *R*_bout_. This value can be determined numerically by computing *R*_bout_ as bout length is varied from one to an implausibly large value and finding the bout length that produces the largest value of *R*_bout_. We used this approach to determine the optimal bout length under a range of reset delays and active-lever–press rates (Fig. 5*C*). Optimal bout length increased with reset delay, according with the well known prediction from foraging theory that optimal patch residence duration increases with the travel time between patches (Stephens and Krebs, 1986). Similarly, optimal bout length increased with the active press rate because working faster yields more rewards over a given duration, even as the ratio requirement increases. Plotting 1-D “slices” through the heat map revealed how optimal bout length (and its associated reward rate) changed as a function of reset delay (with press rate held constant; Fig. 5*D*, left) or press rate (with reset delay held constant; Fig. 5*D*, right).

**Figure 6.**
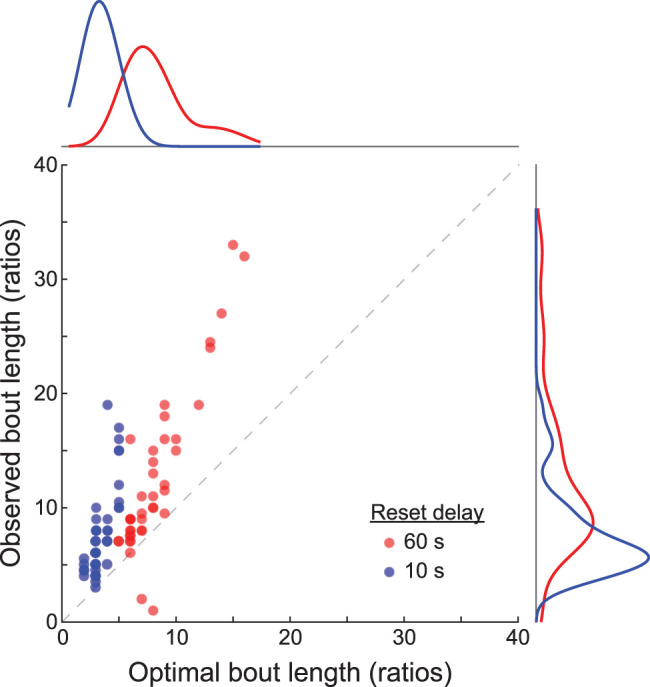
Rats’ strategies were correlated with, but systematically greater than, the optimal bout length. The model-determined optimal bout length is plotted against the median bout length observed in each session. Observed bout length was strongly correlated with the model-predicted optimal bout length. However, nearly all points fall above the dashed unity line, indicating that rats frequently performed longer bouts than predicted by the model. Histograms along the *x*- and *y*-axis show density estimates computed separately for 60 and 10 s reset-delay data.

To compare rats’ behavior to the optimal strategy, we used measured active-lever–press rate and reset-delay length to compute an optimal bout length for each PRR session. While press rate is endogenous to rats’ behavior, we model it here as a trait-like quantity that reflects each rat's preferred tempo of work and ask whether their choice of bout length was normatively matched to it. Figure 6 shows the relationship between optimal bout length and observed bout length for each PRR session. Observed bout length was strongly correlated with optimal bout length (*r*_(87)_ = 0.81; *p* = 9.56 × 10^−22^; Pearson's correlation). Furthermore, the correlation between observed and optimal bout length held when computed separately for PRR-10 sessions (*r*_(47)_ = 0.75; *p* = 1.14 × 10^−9^) and PRR-60 (*r*_(40)_ = 0.89; *p* = 6.94 × 10^−15^) sessions. This means that in addition to being sensitive to the externally imposed reset-delay lengths, rats’ behavior was matched appropriately to the rate of lever pressing they chose to maintain in each session.

### Rats completed “excess ratios” relative to optimal bout length

Although observed bout length correlated strongly with the optimal strategy, inspection of Figure 6 shows that rats were systematically biased toward longer-than-optimal bout lengths. Indeed, in only 2 out of 87 PRR sessions (2.3%), the observed bout length was less than or equal to the optimal bout length. These overly long bouts are reminiscent of the “overharvesting” frequently observed on patch-leaving tasks in a range of species and testing conditions, in which foragers choose patch residence times longer than those predicted by optimal foraging models (Nonacs, 2001; Hayden et al., 2011; Kane et al., 2019; Garcia et al., 2023; Harhen and Bornstein, 2023).

Finally, we considered whether subject-level factors predicted tendency to complete “excess” ratios per bout of work. Two such factors are sex and active-lever–press rate. Indeed, male rats worked a greater number of excess ratios per bout of work than female rats on average (5.65 vs 2.86 excess ratios/bout). Figure 5*A*, however, suggests that sex and active press rate are at least partially correlated, an impression confirmed by a mixed-effect model that found female rats pressed the active lever at significantly lower average rates (Table 14). Thus, to disentangle sex and active press rate, we fit separate mixed-effect models for male and female rats, using the active press rate and reset-delay duration as predictors of excess ratios. In male rats, the active press rate (*β* = 7.33; *p* = 5.95 × 10^−10^), but not reset-delay duration (*β* = 0.60; *p* = 0.15), was a significant predictor of excess ratios (Table 15). The same pattern held in female rats, active press rate (*β* = 10.83, *p* = 4.10 × 10^−3^), but not reset delay (*β* = −0.58, *p* = 0.34), significantly modulated excess ratios (Table 16). Collectively, these analyses suggest that sex modulated the active press rate but that the active press rate predicted tendency to work overly long bouts in both male and female rats.

**Table 14.** A mixed-effect model testing the influence of sex on the PRR active-lever–press rate

Model structure: PRR task active press rate ∼ sex + (1 | Rat ID)					
Effect	Estimate	SE	95% CI		*p*
			LL	UL	
Intercept	0.81	0.09	0.63	0.98	1.89 × 10^−14^
Sex (“female” = 1)	−0.14	0.12	−0.65	−0.16	**1.70 × 10^−3^**

LL, lower limit of confidence interval; UL, upper limit of confidence interval; SE, standard error. Sex was coded as a categorical variable (female = 1). A unique intercept was fit for each subject to account for the repeated-measure structure of the data. Bold *p*-values denote *p* < 0.05.

**Table 15.** A mixed-effect model testing the influence of the active press rate on excess ratios in male rats

Model structure: excess ratios (male rats) ∼ active press rate + reset delay + (1 | Rat ID)					
Effect	Estimate	SE	95% CI		*p*
			LL	UL	
Intercept	−1.22	1.59	−4.42	1.99	0.45
Active press rate	7.33	0.91	5.50	9.16	**5.95 × 10^−10^**
Reset delay (“60 s” = 1)	0.60	0.40	−0.22	1.41	0.15

LL, lower limit of confidence interval; UL, upper limit of confidence interval; SE, standard error. Reset delay was coded as a categorical variable (60 s = 1). A unique intercept was fit for each subject to account for the repeated-measure structure of the data. Bold *p*-values denote *p* < 0.05.

**Table 16.** A mixed-effect model testing the influence of the active press rate on excess ratios in female rats

Model structure: excess ratios (female rats) ∼ active press rate + reset delay + (1 | Rat ID)					
Effect	Estimate	SE	95% CI		*p*
			LL	UL	
Intercept	−1.22	1.59	−4.42	1.99	0.45
Active press rate	10.83	3.56	3.63	18.02	**4.10 × 10^−3^**
Reset delay (“60 s” = 1)	−0.58	0.60	−1.79	0.64	0.34

LL, lower limit of confidence interval; UL, upper limit of confidence interval; SE, standard error. Reset delay was coded as a categorical variable (60 s = 1). A unique intercept was fit for each subject to account for the repeated-measure structure of the data. Bold *p*-values denote *p* < 0.05.

## Discussion

In a typical PR experiment, subjects work on the active lever until they abruptly quit, terminating the testing session. The PRR task introduced here lies on a continuum with this standard approach. Rather than requiring rats to wait until the next testing session for the ratio to reset, PRR offers subjects the opportunity to reset the ratio at the expense of a much shorter reset delay and gives them agency over when ratio resets occur. This allows subjects to make multiple “microquitting” decisions within each session rather than committing to a single, irreversible decision to quit. PRR thus enables more accurate, trial-level estimation of how subjects weigh effort and delay costs instead of each session culminating in a single, point estimate of quitting threshold. We found that breakpoint on standard PR was strongly correlated with bout length on PRR, suggesting that the PRR taps into similar forms of decision-making as standard PR but with improved sensitivity that comes from having multiple trials per session for analysis. These more fine-grained features of the PRR task will make it more amenable to the study of the pharmacological modulation of decision trade-offs, as well as the neural and computational mechanisms of cost-sensitivity phenotypes.

Previous studies have explored the addition of ratio resets to PR schedules. For instance, Hurwitz and Harzem (1968) tested rats on a PR task that included a cost-free ratio reset option and found that rats typically reset the ratio after earning one or at most a few rewards. This strategy makes economic sense, as it held effort costs low, and there was no penalty associated with resetting the ratio frequently. However, arriving at this solution required little weighing of cost and benefit by rats, as resetting after every reward was likely the optimal strategy in all the cases that Hurwitz and Harzem (1968) tested. Dardano (1974) offered pigeons three concurrent choice options: one key produced food reward on a steep PR schedule, a second key reset the ratio but also caused a shock, while a third key resulted in a 3 min time-out during which pecks on any key were ignored. Pigeons reset the ratio frequently when shock intensity was low but increased their selection of the time-out option as shock intensity increased. This procedure effectively measured subjects’ relative aversion to effort costs and physical pain. However, the results are difficult to interpret in an economic framework, as selection of the time-out option was never advantageous and may have reflected frustration with the effort and pain associated with the other options.

In contrast, results of the PRR task allow an economically interpretable dissociation of temporal preferences and effort sensitivity. For instance, a rat that resets the ratio frequently reveals a level of delay tolerance that can be quantified precisely by observing its behavior under different reset-delay conditions. Similarly, a delay-averse rat with low-effort sensitivity would show a different phenotype, instead of resetting infrequently and running the ratio up to higher levels with each bout of work. Neuropsychiatric disorders are frequently associated with alterations in sensitivity to time and effort costs, each related to preclinical features of impulsivity (Evenden, 1999; Dalley et al., 2004; Winstanley and Floresco, 2016) and anergia (Salamone et al., 1994, 2022; Treadway et al., 2009; Treadway and Salamone, 2022; Salamone and Correa, 2024), respectively. The PRR task offers a way of dissociating the impact of these constructs using a single behavioral approach.

The PRR task also represents a conceptual bridge between distinct approaches to studying decision-making, combining the behaviorist heritage of the PR schedule with the more naturalistic decision topology favored by behavioral ecologists. Indeed, rats’ PRR behavior aligned with both of these perspectives. As noted above, the correlation between PR breakpoint and PRR bout length suggests the tasks measure similar cost-sensitivity constructs. At the same time, the application of normative, foraging theory inspired modeling to the PRR task revealed a behavioral signature familiar to students of the patch-leaving framework: overharvesting, which manifested as a reluctance to reset the ratio as frequently as the reward-maximizing strategy predicted. Similar overharvesting has been observed on patch-leaving tasks, and understanding its causes is an active area of research (Constantino and Daw, 2015; Cash-Padgett and Hayden, 2020; Kendall and Wikenheiser, 2022; Doren et al., 2023; Gancarz et al., 2023; Harhen and Bornstein, 2023; Lin and von Helversen, 2023). The PRR task offers another means of tackling this question by forging a deep connection between complementary approaches to studying animal behavior. Despite the many strengths of the present PRR task design, the randomization of conditions we administered here is not ideal for studying how rats’ strategies may change with experience. Investigating how strategies evolve over time on task is an important direction for future work.

The normative modeling framework used here makes PRR a new tool for studying persistence in pursuit of a goal versus disengagement from it (Shidara et al., 1998; La Camera and Richmond, 2008; Inaba et al., 2013; Regalado et al., 2024; Vazquez et al., 2024; Aenugu and O'Doherty, 2025; Gonzalez et al., 2025), a question which is also relevant to our understanding of neuropsychiatric conditions. However, it is important to note that while our modeling approach provides a normative yardstick for assessing behavior, it does not speak to the neural mechanisms of decision-making, which represents a next step. A process-level model, based on ideas of effort and temporal discounting, would aid the development of mechanistic hypotheses for how subjects solve the task and may be useful for translational progress.

## Data Availability

All data and analysis code are freely available at https://zenodo.org/records/15577773.

## Synthesis

Reviewing Editor: Adrien Peyrache, McGill University Decisions are customarily a result of the Reviewing Editor and the peer reviewers coming together and discussing their recommendations until a consensus is reached. When revisions are invited, a fact-based synthesis statement explaining their decision and outlining what is needed to prepare a revision will be listed below. The following reviewer(s) agreed to reveal their identity: John Salamone, Stan Floresco.

This study represents a potentially important contribution to the literature and may guide future research on the effects of pharmacological or other neural manipulations. The reviewers have raised several points that should be addressed to render the manuscript suitable for publication. Their main comments are outlined below.

Reviewer #1

On p 25, the authors cite the Salamone et al. (1994) paper ostensibly as an example of a study focusing on 'anhedonia'. However, that paper and other related papers showed clearly that the effort-related impairments induced by DA antagonism or DA depletion are not related to the induction of 'anhedonia'. The title of this paper began with the phrase 'Anhedonia or anergia?', and the very firm conclusion was that the data did not at all fit the definition of 'anhedonia', but rather, was better described as anergia. In terms of the animal procedures and result reviewed in the Treadway and Salamone (2022) review, the conclusion was again very clear that the data are inconsistent with the use of the term 'anhedonia'. In fact, all the papers from the Salamone lab cited in the present manuscript provide the same conclusion. The specific and original definition of anhedonia is 'the inability to experience pleasure' (e.g. Ribot), and there is no evidence that alterations in effort-based decision making that result from impaired DA transmission are due to a lack of hedonic reactivity. That conclusion is consistent with the large body of research from Berridge, Robinson and colleagues, and is reviewed very thoroughly in papers such as Salamone et al. (2022; Brain Research Reviews) or Salamone and Correa (2024; Annual Review of Psychology). This conclusion is also supported by papers that use the concurrent progressive ratio/chow feeding choice procedure (e.g. Randall et al., 2012, 2014). Considerable evidence indicates that dopaminergic manipulations that alter effort-based choice produce effects that are independent of actions on primary food motivation, the unconditional reinforcing properties of food, hedonic reactivity to food, hedonic eating of highly palatable food, food preference, sucrose preference, etc. Thus, it is a mistake to consider that studies on the effects of dopaminergic manipulations on effort-based choice in any way reflect 'anhedonia'. The present manuscript offers a potentially important novel behavioral procedure, and the overall review of the behavior analysis literature is excellent. Nevertheless, this reference to 'anhedonia' needs to be corrected in the present manuscript.

Reviewer #2

In general, the study is well-conducted and analyzed appropriately. I do find it somewhat curious that no neural/pharmacological manipulations were included here (e.g.; how might a dopamine antagonist affect these measures, considering how key dopamine is to effort related behaviors). Nevertheless, there is utility in this study in that it sets the foundation for future studies of this kind. That said, whenever a new task is being described, there are key details that should be included that will help others who wish to use this task implement these type of manipulations.

Issues to be addressed (in order of appearance in the manuscript)

1) The authors note that for the PR task, when a reward was delivered a light was illuminated above it for a period (and the lever was inactive). How long was this light on/lever inactive?

2) The way the methods are written, after going through pretraining, rats transition to training on one of the 3 tasks on separate days. What is unclear is, for the data analyzed here, how many total days of training were rats given? One of the figure captions implies the data were taken from one session, but was that the 1st session, or after animals had more extensive experience with the 3 tasks. Were all the data analyzed taken from a single session, or was it collapsed across sessions? If the latter, how many training sessions were included and from what part of training.

3) Relatedly, it would be important to show if there are changes in rewards earned/reset presses over the course of training. I suspect that this might change over time with experience on the task, but it is unclear. Does it take a while for rats to reach a steady state of responding across the tasks? This should be addressed and some data on this should be presented.

## References

[B1] Aberman JE, Ward SJ, Salamone JD (1998) Effects of dopamine antagonists and accumbens dopamine depletions on time-constrained progressive-ratio performance. Pharmacol Biochem Behav 61:341–348. 10.1016/s0091-3057(98)00112-9 9802826

[B2] Aenugu S, O'Doherty JP (2025) Building momentum: a computational account of persistence toward long-term goals. PLoS Comput Biol 21:e1013054. 10.1371/journal.pcbi.1013054 40367239 PMC12101773

[B3] Cagniard B, Beeler JA, Britt JP, McGehee DS, Marinelli M, Zhuang X (2006) Dopamine scales performance in the absence of new learning. Neuron 51:541–547. 10.1016/j.neuron.2006.07.026 16950153

[B4] Cash-Padgett T, Hayden B (2020) Behavioural variability contributes to over-staying in patchy foraging. Biol Lett 16:20190915. 10.1098/rsbl.2019.0915 32156171 PMC7115184

[B5] Charnov EL (1976) Optimal foraging, the marginal value theorem. Theor Popul Biol 9:129–136. 10.1016/0040-5809(76)90040-x 1273796

[B6] Chen Y, Breitborde NJ, Peruggia M, Van Zandt T (2022) Understanding motivation with the progressive ratio task: a hierarchical Bayesian model. Comput Brain Behav 5:81–102. 10.1007/s42113-021-00114-1

[B7] Constantino SM, Daw ND (2015) Learning the opportunity cost of time in a patch-foraging task. Cogn Affect Behav Neurosci 15:837–853. 10.3758/s13415-015-0350-y 25917000 PMC4624618

[B8] Dalley JW, Cardinal RN, Robbins TW (2004) Prefrontal executive and cognitive functions in rodents: neural and neurochemical substrates. Neurosci Biobehav Rev 28:771–784. 10.1016/j.neubiorev.2004.09.006 15555683

[B9] Dardano JF (1974) Response-produced timeouts under a progressive-ratio schedule with a punished reset option. J Exp Anal Behav 22:103–113. 10.1901/jeab.1974.22-103 16811767 PMC1333246

[B10] Doren N, Chung HK, Grueschow M, Quednow BB, Hayward-Könnecke H, Jetter A, Tobler PN (2023) Acetylcholine and noradrenaline enhance foraging optimality in humans. Proc Natl Acad Sci U S A 120:e2305596120. 10.1073/pnas.2305596120 37639601 PMC10483619

[B11] Emlen JM (1968) Optimal choice in animals. Am Nat 102:385–389. 10.1086/282551

[B12] Evenden J (1999) Impulsivity: a discussion of clinical and experimental findings. J Psychopharmacol 13:180–192. 10.1177/026988119901300211 10475725

[B13] Gancarz AM, et al. (2023) Reward maximization assessed using a sequential patch depletion task in a large sample of heterogeneous stock rats. Sci Rep 13:7027. 10.1038/s41598-023-34179-8 37120610 PMC10148848

[B14] Garcia M, Gupta S, Wikenheiser AM (2023) Sex differences in patch-leaving foraging decisions in rats. Oxf Open Neurosci 2:kvad011. 10.1093/oons/kvad011 38596244 PMC11003400

[B15] Gonzalez VV, Malvaez M, Yeghikian A, Wissing S, Sharpe M, Wassum KM, Izquierdo A (2025) A common stay-on-goal mechanism in the anterior cingulate cortex for information and effort choices. eNeuro 12:ENEURO.0454-24.2025. 10.1523/ENEURO.0454-24.2025 PMC11964290 39947906

[B16] Griffiths RR, Thompson T (1973) The post-reinforcement pause: a misnomer. Psychol Rec 23:229–235. 10.1007/BF03394160

[B17] Harhen NC, Bornstein AM (2023) Overharvesting in human patch foraging reflects rational structure learning and adaptive planning. Proc Natl Acad Sci U S A 120:e2216524120. 10.1073/pnas.2216524120 36961923 PMC10068834

[B21] Hart EE, Izquierdo A (2019) Quantity versus quality: convergent findings in effort-based choice tasks. Behav Processes 164:178–185. 10.1016/j.beproc.2019.05.009 31082477 PMC6557689

[B18] Hart EE, Gerson JO, Zoken Y, Garcia M, Izquierdo A (2017) Anterior cingulate cortex supports effort allocation towards a qualitatively preferred option. Eur J Neurosci 46:1682–1688. 10.1111/ejn.13608 28543944 PMC6442740

[B19] Hart EE, Gerson JO, Izquierdo A (2018) Persistent effect of withdrawal from intravenous methamphetamine self-administration on brain activation and behavioral economic indices involving an effort cost. Neuropharmacology 140:130–138. 10.1016/j.neuropharm.2018.07.023 30053443 PMC6442736

[B20] Hart EE, Blair GJ, O'Dell TJ, Blair HT, Izquierdo A (2020) Chemogenetic modulation and single-photon calcium imaging in anterior cingulate cortex reveal a mechanism for effort-based decisions. J Neurosci 40:5628–5643. 10.1523/JNEUROSCI.2548-19.2020 32527984 PMC7363467

[B22] Hayden BY, Pearson JM, Platt ML (2011) Neuronal basis of sequential foraging decisions in a patchy environment. Nat Neurosci 14:933–939. 10.1038/nn.2856 21642973 PMC3553855

[B23] Hodos W (1961) Progressive ratio as a measure of reward strength. Science 134:943–944. 10.1126/science.134.3483.943 13714876

[B24] Hurwitz HM, Harzem P (1968) Progressive ratio performance with reset option. Psychol Rec 18:553–558. 10.1007/BF03393806

[B25] Inaba K, Mizuhiki T, Setogawa T, Toda K, Richmond BJ, Shidara M (2013) Neurons in monkey dorsal raphe nucleus code beginning and progress of step-by-step schedule, reward expectation, and amount of reward outcome in the reward schedule task. J Neurosci 33:3477–3491. 10.1523/JNEUROSCI.4388-12.2013 23426675 PMC6619532

[B26] Jerome J, Sturmey P (2008) Reinforcing efficacy of interactions with preferred and nonpreferred staff under progressive-ratio schedules. J Appl Behav Anal 41:221–225. 10.1901/jaba.2008.41-221 18595285 PMC2408338

[B27] Kane GA, Bornstein AM, Shenhav A, Wilson RC, Daw ND, Cohen JD (2019) Rats exhibit similar biases in foraging and intertemporal choice tasks. Elife 8:e48429. 10.7554/eLife.48429 31532391 PMC6794087

[B28] Kendall RK, Wikenheiser AM (2022) Quitting while you're ahead: patch foraging and temporal cognition. Behav Neurosci 136:467–478. 10.1037/bne0000526 35834190

[B29] Killeen PR, Posadas-Sanchez D, Johansen EB, Thrailkill EA (2009) Progressive ratio schedules of reinforcement. J Exp Psychol Anim Behav Process 35:35–50. 10.1037/a0012497 19159161 PMC2806234

[B30] La Camera G, Richmond BJ (2008) Modeling the violation of reward maximization and invariance in reinforcement schedules. PLoS Comput Biol 4:e1000131. 10.1371/journal.pcbi.1000131 18688266 PMC2453237

[B31] Lendrem D (2012) *Modelling in behavioural ecology: an introductory text*. Springer Science & Business Media.

[B32] Lin HY, von Helversen B (2023) Never gonna give you up even when it is suboptimal. Cogn Sci 47:e13323. 10.1111/cogs.13323 37486808

[B33] MacArthur RH, Pianka ER (1966) On optimal use of a patchy environment. Am Nat 100:603–609. 10.1086/282454

[B34] Nonacs P (2001) State dependent behavior and the marginal value theorem. Behav Ecol 12:71–83. 10.1093/oxfordjournals.beheco.a000381

[B35] Regalado JM, Corredera Asensio A, Haunold T, Toader AC, Li YR, Neal LA, Rajasethupathy P (2024) Neural activity ramps in frontal cortex signal extended motivation during learning. Elife 13:RP93983. 10.7554/eLife.93983 39037775 PMC11262795

[B39] Salamone JD, Correa M (2024) The neurobiology of activational aspects of motivation: exertion of effort, effort-based decision making, and the role of dopamine. Annu Rev Psychol 75:1–32. 10.1146/annurev-psych-020223-012208 37788571

[B36] Salamone JD, Steinpreis RE, McCullough LD, Smith P, Grebel D, Mahan K (1991) Haloperidol and nucleus accumbens dopamine depletion suppress lever pressing for food but increase free food consumption in a novel food choice procedure. Psychopharmacology 104:515–521. 10.1007/BF02245659 1780422

[B37] Salamone JD, Cousins MS, Bucher S (1994) Anhedonia or anergia? Effects of haloperidol and nucleus accumbens dopamine depletion on instrumental response selection in a T-maze cost/benefit procedure. Behav Brain Res 65:221–229. 10.1016/0166-4328(94)90108-2 7718155

[B38] Salamone JD, et al. (2022) Complexities and paradoxes in understanding the role of dopamine in incentive motivation and instrumental action: exertion of effort vs. anhedonia. Brain Res Bull 182:57–66. 10.1016/j.brainresbull.2022.01.019 35151797

[B40] Schoener TW (1987) *A brief history of optimal foraging ecology*. Boston, MA: Springer US.

[B41] Shidara M, Aigner TG, Richmond BJ (1998) Neuronal signals in the monkey ventral striatum related to progress through a predictable series of trials. J Neurosci 18:2613–2625. 10.1523/JNEUROSCI.18-07-02613.1998 9502820 PMC6793099

[B42] Skinner BF (2019) *The behavior of organisms: an experimental analysis*. Cambridge: BF Skinner Foundation.

[B43] Stafford D, LeSage MG, Glowa JR (1998) Progressive-ratio schedules of drug delivery in the analysis of drug self-administration: a review. Psychopharmacology 139:169–184. 10.1007/s002130050702 9784071

[B44] Stephens DW (2008) Decision ecology: foraging and the ecology of animal decision making. Cogn Affect Behav Neurosci 8:475–484. 10.3758/CABN.8.4.475 19033242

[B45] Stephens DW, Krebs JR (1986) *Foraging theory*. Princeton, New Jersey: Princeton University Press.

[B47] Treadway MT, Salamone JD (2022) Vigor, effort-related aspects of motivation and anhedonia. Curr Top Behav Neurosci 58:325–353. 10.1007/7854_2022_355 35505057

[B46] Treadway MT, Buckholtz JW, Schwartzman AN, Lambert WE, Zald DH (2009) Worth the ‘EEfRT'? The effort expenditure for rewards task as an objective measure of motivation and anhedonia. PLoS One 4:e6598. 10.1371/journal.pone.0006598 19672310 PMC2720457

[B48] Vazquez D, Maulhardt SR, Stalnaker TA, Solway A, Charpentier CJ, Roesch MR (2024) Optogenetic inhibition of rat anterior cingulate cortex impairs the ability to initiate and stay on task. J Neurosci 44:e1850232024. 10.1523/JNEUROSCI.1850-23.2024 38569923 PMC11097287

[B49] Winstanley CA, Floresco SB (2016) Deciphering decision making: variation in animal models of effort- and uncertainty-based choice reveals distinct neural circuitries underlying core cognitive processes. J Neurosci 36:12069–12079. 10.1523/JNEUROSCI.1713-16.2016 27903717 PMC6601981

[B50] Yohn SE, Santerre JL, Nunes EJ, Kozak R, Podurgiel SJ, Correa M, Salamone JD (2015) The role of dopamine D1 receptor transmission in effort-related choice behavior: effects of D1 agonists. Pharmacol Biochem Behav 135:217–226. 10.1016/j.pbb.2015.05.003 26022661

[B51] Yu Z, Guindani M, Grieco SF, Chen L, Holmes TC, Xu X (2022) Beyond t test and ANOVA: applications of mixed-effects models for more rigorous statistical analysis in neuroscience research. Neuron 110:21–35. 10.1016/j.neuron.2021.10.030 34784504 PMC8763600

