# Supplementary figures and images for: A Progressive Ratio Task with Costly Resets Reveals Adaptive Effort-Delay Trade-Offs
Source: eNeuro. 2025 Nov 12;12(11):ENEURO.0258-25.2025. doi: 10.1523/ENEURO.0258-25.2025 (PMC12618050; doi:10.1523/ENEURO.0258-25.2025)

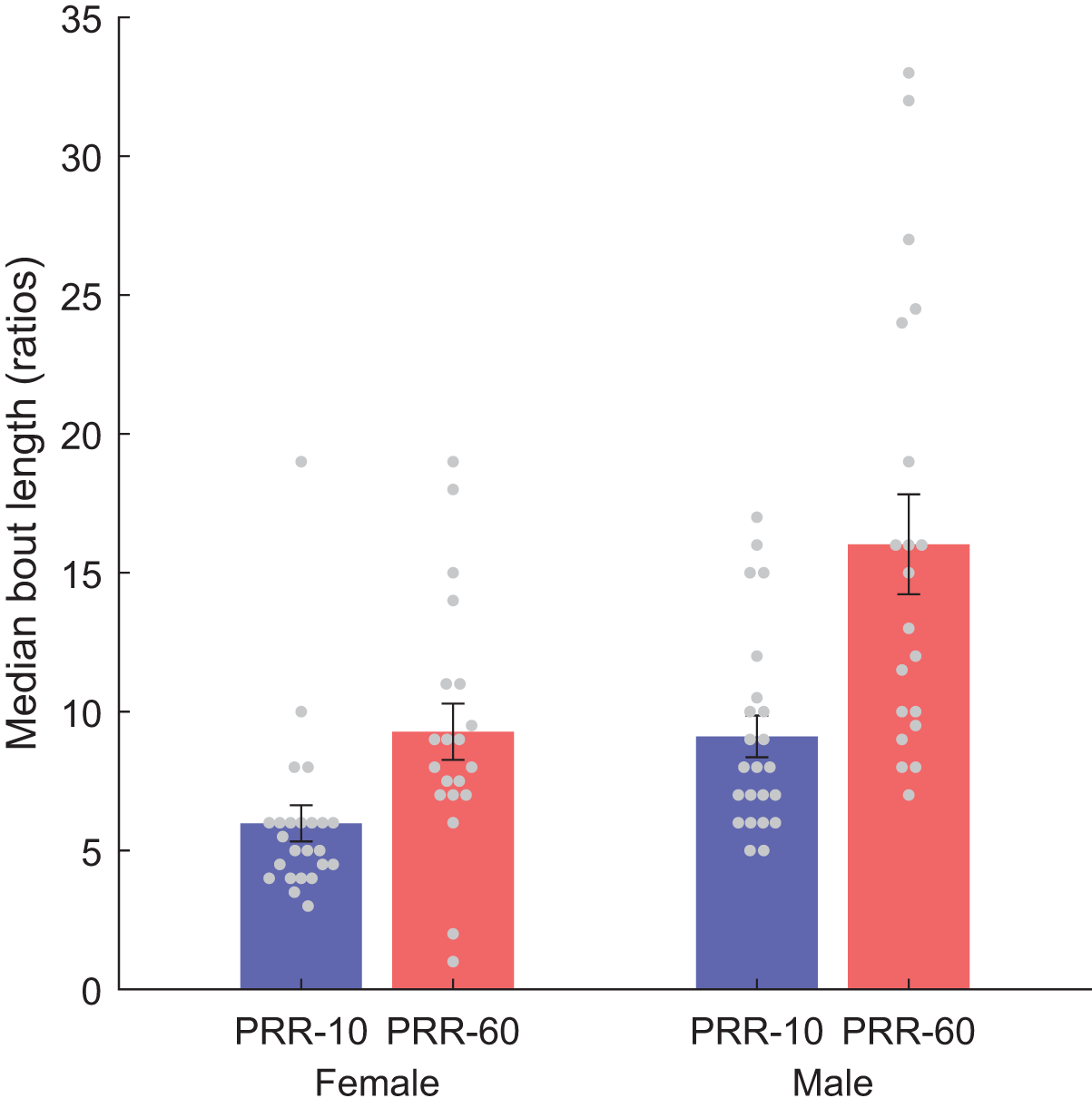

Supplement: Figure 4-1 — Reset delay affected bout length differently in male and female rats. Median bout length for PRR sessions is plotted separately for male and female rats. Each point indicates the median bout length in one session, and error bars indicated the mean ± SEM. General patterns are similar across male and female rats with longer reset delays eliciting longer bouts of work. However, this effect was strong in male rats, leading to a significant interaction between sex and reset delay (Table 8). Download Figure 4-1, TIF file. [file eneuro-12-ENEURO.0258-25.2025-s002.tif]
